# Computational Characteristics of the Striatal Dopamine System Described by Reinforcement Learning With Fast Generalization

**DOI:** 10.3389/fncom.2020.00066

**Published:** 2020-07-22

**Authors:** Yoshihisa Fujita, Sho Yagishita, Haruo Kasai, Shin Ishii

**Affiliations:** ^1^Integrated Systems Biology Laboratory, Department of Systems Science, Graduate School of Informatics, Kyoto University, Kyoto, Japan; ^2^Laboratory of Structural Physiology, Center for Disease Biology and Integrative Medicine, Faculty of Medicine, The University of Tokyo, Tokyo, Japan; ^3^International Research Center for Neurointelligence, The University of Tokyo Institutes for Advanced Study, The University of Tokyo, Tokyo, Japan; ^4^Neural Information Processing Laboratories, Advanced Telecommunications Research Institute International (ATR), Kyoto, Japan

**Keywords:** generalization, adaptive behaviors, reward learning, striatum, dopamine-dependent plasticity, reinforcement learning, artificial neural networks

## Abstract

Generalization is the ability to apply past experience to similar but non-identical situations. It not only affects stimulus-outcome relationships, as observed in conditioning experiments, but may also be essential for adaptive behaviors, which involve the interaction between individuals and their environment. Computational modeling could potentially clarify the effect of generalization on adaptive behaviors and how this effect emerges from the underlying computation. Recent neurobiological observation indicated that the striatal dopamine system achieves generalization and subsequent discrimination by updating the corticostriatal synaptic connections in differential response to reward and punishment. In this study, we analyzed how computational characteristics in this neurobiological system affects adaptive behaviors. We proposed a novel reinforcement learning model with multilayer neural networks in which the synaptic weights of only the last layer are updated according to the prediction error. We set fixed connections between the input and hidden layers to maintain the similarity of inputs in the hidden-layer representation. This network enabled fast generalization of reward and punishment learning, and thereby facilitated safe and efficient exploration of spatial navigation tasks. Notably, it demonstrated a quick reward approach and efficient punishment aversion in the early learning phase, compared to algorithms that do not show generalization. However, disturbance of the network that causes noisy generalization and impaired discrimination induced maladaptive valuation. These results suggested the advantage and potential drawback of computation by the striatal dopamine system with regard to adaptive behaviors.

## Introduction

Animals' survival incorporates reward-seeking behavior accompanied by risks. Outcome observation resulting from the pairing of a current state and a taken action provide clues to ensure optimal behaviors, but it may be associated with substantial energy consumption and aversive experiences. Such a learning process is inefficient and even harmful, especially when animals are required to adapt to new environments. Animals instead generalize their previous experiences to predict outcome, even in novel situations. If the prediction due to generalization is different from the actual observation, the prediction is then reshaped by discrimination learning. Generalization and discrimination may be essential for efficient adaptive behaviors, whereas abnormalities in these functions can be maladaptive. Generalization to an abnormal extent has been implicated in psychiatric disorders (Dunsmoor and Paz, 2015; Kahnt and Tobler, 2016; Asok et al., 2018). A recent neurobiological study presented the possibility that disrupted discrimination is involved in psychotic symptoms (Iino et al., 2020).

Psychological studies have investigated generalization by using the conditioning paradigm in behavioral experiments (Ghirlanda and Enquist, 2003). If a response has been established by a stimulus paired with an outcome (i.e., reward or punishment), then resembling stimuli evoke similar responses. This “law of effect” depends on the extent that the second stimulus resembles the first stimulus, and is termed “stimulus generalization” (Thorndike, 1898; Ghirlanda and Enquist, 2003). Discrimination can then occur if the first stimulus is paired with a reward but the resembling stimulus is paired with no reward; as a consequence, only the first stimulus elicits a response. How the brain establishes stimulus generalization has been explained, based on artificial neural networks (Shepard and Kannappan, 1991; Ghirlanda and Enquist, 1998; Franks and Ruxton, 2008; Wisniewski et al., 2012). These previous studies have focused on the stimulus–response relationship, although generalization can be incorporated in the interaction between individuals and the environment. Reinforcement learning involves this interaction and is used as a model of reward-driven learning (Frank et al., 2004; Doya, 2007; Glimcher, 2011). In the field of artificial intelligence, reinforcement learning in combination with artificial neural networks achieves a high performance, which suggests the contribution of generalization to adaptive behaviors (Mnih et al., 2015). However, neurobiological evidence indicates that the neural system has a unique computation for reward-driven learning and generalization, compared to algorithms used for artificial intelligence (Whittington and Bogacz, 2019). This raises the question regarding how its computational characteristics differ from those of ordinary algorithms. Advantages and shortcomings should exist with regard to adaptive behaviors.

Theoretical attempts have focused on separate systems that process positive and negative values in the brain (DeLong, 1990; Nambu, 2007; Amemori et al., 2011; Collins and Frank, 2014), which are not adopted when using ordinary reinforcement learning. Dopamine and its main target area, the striatum, play central roles in reward- and punishment-related learning (Meredith et al., 2008). Dopaminergic neurons show a positive response to a greater-than-expected reward and a negative response to a less-than-expected reward, which indicates that dopamine codes reward prediction error (Schultz, 2015). The striatum receives dopamine signals and glutamatergic input from the cortex and thalamus (Tepper et al., 2007). Dopamine modulates synaptic plasticity between the cortex and the striatum during reward-related learning (Reynolds et al., 2001). The striatum is primarily composed of spiny projection neurons (SPNs), which can be divided into SPNs that primarily express the dopamine D1 receptor (D1-SPNs) and SPNs that express the dopamine D2 receptor (D2-SPNs) (Surmeier et al., 2010). D1-SPNs respond to phasic increases in dopamine (Yagishita et al., 2014), whereas D2-SPNs respond to the phasic decreases in dopamine (Hikida et al., 2013; Iino et al., 2020). Perturbing the activity of D1- and D2-SPNs inhibits reward learning and punishment learning, respectively (Hikida et al., 2013). The advantages of having such separate systems have been discussed in computational studies (Mikhael and Bogacz, 2016; Elfwing and Seymour, 2017). One study (Mikhael and Bogacz, 2016) demonstrated an advantage in learning reward uncertainty. Another study (Elfwing and Seymour, 2017) showed the possibility of achieving safe behaviors.

However, recent neural recordings and optogenetic manipulations provide some data that suggest different roles of SPNs from those in the existing models (Cox and Witten, 2019). Our recent experiments with a classical conditioning task found that D1- and D2-SPNs are differentially responsible for stimulus generalization and discrimination (Iino et al., 2020). The same series of experiments revealed that stimulus generalization/discrimination occurred solely by dopamine-dependent plasticity of SPN spines that receive cortical inputs, which implies that updating in other connections (e.g., intracortical synaptic connections) would be minor. These observations suggest learning rules that update the synaptic weights of the last layer in a multilayer neural network—in our case, corticostriatal connections—are essential. Such learning rules actually enable remarkably fast learning in reservoir computing (RC) and extreme learning machine (ELM) (Maass et al., 2002; Huang et al., 2006; Lukoševičius and Jaeger, 2009). RC and ELM are neural networks that train only their readouts (i.e., the last layer connections); RC has recurrent connections, whereas ELM does not. Iino et al. also showed that administration of methamphetamine, which causes psychosis (e.g., delusions) in humans, impaired discrimination function in mice by altering dopamine dynamics associated with unexpected reward omission (Iino et al., 2020). Taken together with the physiological functions of D2-SPN described above, these findings suggest that impairment of dopamine-dependent corticostriatal plasticity of D2-SPNs can induce abnormal value prediction via disrupted discrimination.

In this study, we propose a novel reinforcement learning model that reproduces stimulus generalization and discrimination, while accounting for the physiological characteristics of striatal SPNs. We used a neural network for value estimation and introduced fixed connections between the input and hidden layers, as in RC and ELM. RC has the potential for context-dependent value estimation because of its recurrent connections. However, for simplicity, we adopted a feed-forward neural network with the same architecture as ELM. The extent of stimulus generalization depends on the similarity of stimuli; therefore, we did not use random and fixed connections as implemented in ordinary ELM. We instead set the fixed connections so as to maintain the similarity of inputs in the hidden-layer representation. In addition, we introduced connections that had weights separately updated by positive and negative reward prediction errors, while taking into account the differential roles of D1- and D2-SPNs in the striatum. This neural network enabled generalization in a quick manner. We named this model “Outspread Valuation for Reward Learning and Punishment Learning” (“OVaRLAP”). Using this model for painful grid-world navigation tasks, we first evaluated the contribution of generalization and discrimination to adaptive behaviors. The OVaRLAP model performed safe and efficient exploration, suggesting that quick generalization of punishment learning contributed to safe and efficient reward-seeking and pain-avoiding. We then tested disturbed OVaRLAP in a painless grid-world to examine whether abnormal generalization and discrimination underlies maladaptive behaviors, as implied in psychological and psychiatric studies (Buss and Daniell, 1967; Ralph, 1968; Kahnt and Tobler, 2016). We introduced impairment of learning from negative prediction error, which disables discrimination, based on the physiological findings (Iino et al., 2020). We found that impaired discrimination combined with noisy generalization induced aberrant valuation, after repeating reward-seeking behaviors. These results showed that the unique computation suggested by the striatal dopamine system facilitated safe and efficient exploration, but on the other hand had potential defects which can cause maladaptive behaviors.

## Methods

**Figure 1:**
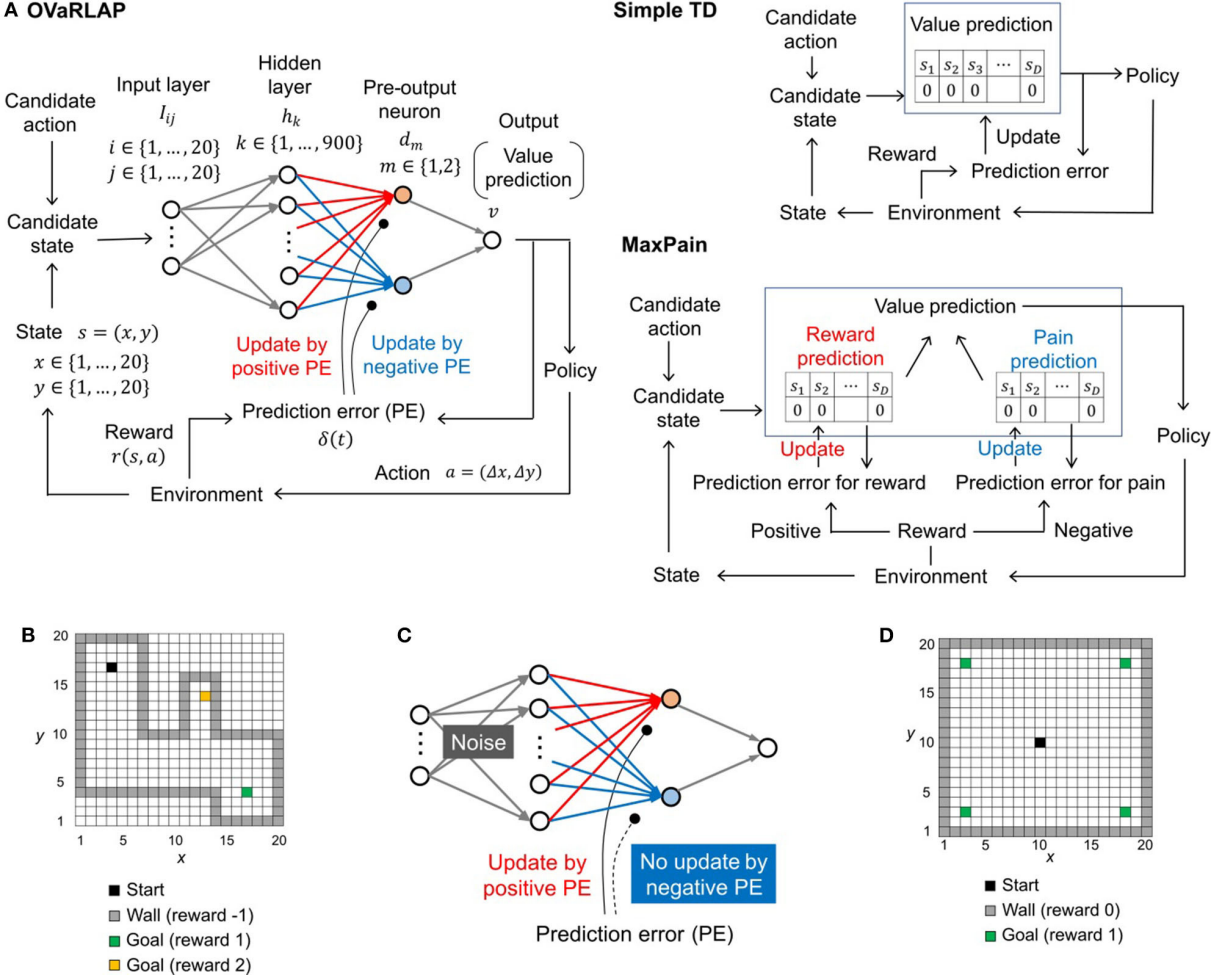
The architecture of TD learning algorithms and the task. **(A)** Schematic diagrams of OVaRLAP (left), simple TD learning (middle), and MaxPain (right). In OVaRLAP, the value prediction used a neural network with fixed connections (gray arrows) and distinct connections updated by positive and negative TD errors (red and blue arrows, respectively). In our implementations of simple TD learning and MaxPain, the predicted value is represented by look-up tables in which the state, *s*_*k*_, indicates a position (*x, y*) in the two-dimensional grid-world. **(B)** The navigation task in the two-dimensional grid-world. The non-gray squares and gray squares indicate passable and not passable, respectively. The black square is the starting position, and the green and yellow squares are the goals at which the agent receives the positive rewards of 1 and 2, respectively. When the agent receives a positive reward, an episode ends so that the agent restarts the task from the starting position. If an agent hits a wall (i.e., a not passable square), it receives a negative reward of −1, but continues the task by staying at the same square. **(C)** Schematic diagram of OVaRLAP in which updating connections, based on the negative TD error (blue connections), are impaired. In addition, noise is introduced to induce an anomaly in the initialization of the fixed connections between the input and the hidden layers (for the details of noise here, see Section Model Description of OVaRLAP). **(D)** The navigation task in the two-dimensional grid-world. The black square is the starting position. The green squares are the goals at which the agent receives a positive reward of one. If the agent receives a positive reward, a single episode ends, and a new episode restarts from the starting position. In this task, when the agent hits a wall (i.e., a gray square), no negative reward is given, and the agent remains at the same square.

We developed OVaRLAP to analyze how the computational characteristics in the striatal dopamine system affect behaviors. We first evaluated OVaRLAP in a spatial navigation task in painful grid-worlds by comparing its performance with those of two other representative algorithms (Figures 1A,B). We then examined the behavior of disturbed OVaRLAP in a spatial navigation task in a painless grid-world (Figures 1C,D).

### Model Description of OVaRLAP

A neural network was used for value estimation in OVaRLAP. It consists of an input layer, a hidden layer, two pre-output neurons, and an output neuron (Figure 1A, left). The input represents the state at discretized time, *t*. We applied our learning method to two types of navigation tasks, both in two-dimensional grid-worlds, in which position (*x, y*), *x* ∈ {1, …, 20}, and *y* ∈ {1, …20} were simply represented by a two-dimensional index function:

$$\begin{array}{l} I_{ij}\left(x,y,t\right)=\left\{ \begin{array}{c} 1,\text{if }i=x\text{ and }j=y\\ \;0,\text{ otherwise}, \end{array}\right. \end{array}$$

for *i* = 1, …, 20 and *j* = 1, …, 20.

The input signals were transformed by fixed connections into hidden-layer activity patterns. We set the number of hidden-layer neurons and the weight of fixed connections so as to reproduce the shape of the generalization gradient, which has been commonly observed across various species, behavioral contexts, and sensory modalities (Ghirlanda and Enquist, 2003). The number of hidden-layer neurons was set to 900. The hidden-layer activity *h*_*k*_(*x, y, t*) for *k* = 1, …, 900 was given by

$$\begin{array}{l} h_{k}\left(x,y,t\right)\;=\;\underset{i}{\sum }\underset{j}{\sum }M_{ij,k}I_{ij}\left(x,y,t\right) \end{array}$$

in which *M*_*ij, k*_ is the weight of the fixed connection from the input-layer neuron (*i, j*) to the hidden-layer neuron *k*. Based on the definition of *I*_*ij*_(*x, y, t*), *h*_*k*_(*x, y, t*) was also represented as

$$\begin{array}{l} h_{k}\left(x,y,t\right)\;=\;M_{xy,k} \end{array}$$

in which (*x, y*) is the agent's position at time *t*.

For the generalization capability, we assumed the hidden-layer representation becomes similar when the input signal is similar. In our case, the values of *h*_*k*_(*x*_1_, *y*_1_, *t*) and *h*_*k*_(*x*_2_, *y*_2_, *t*) are similar if position (*x*_1_, *y*_1_) is close to position (*x*_2_, *y*_2_), based on Euclidian distance. To reflect this request, we set the fixed connections *M*_*ij, k*_ to follow the two-dimensional Gaussian function:

$$\begin{array}{l} M_{ij,k}=\frac{\exp\left\{-\left(\frac{{\left(i-a_{k}\right)}^{2}}{2\sigma_{k}^{2}}+\frac{{\left(j-b_{k}\right)}^{2}}{2\sigma_{k}^{2}}\right)\right\}+\varepsilon_{ij,k}}{D} \end{array}$$

in which *D* is the number of the input-layer neurons used for normalization (*D* = 400, because of the 20 × 20 grid-worlds); *a*_*k*_ and *b*_*k*_ denote the center and are set as *a*_*k*_ ∈ {1, …, 20}, *b*_*k*_ ∈ {1, …, 20}, and 20(*a*_*k*_ − 1) + *b*_*k*_ = *ceil*(400*k*/900) so that the central location **(***a*_*k*_, *b*_*k*_**)** in the 20 × 20 2D space was linearly correlated with the location $({\tilde{a}}_{k},{\tilde{b}}_{k})$ in 30 × 30 2D space where ã_*k*_ = 1 + *floor*((*k* − 1)/30) and ${\tilde{b}}_{k}=mod\left(k-1,\;30\right)+1$; $\sigma_{k}^{2}$ is the variance sampled from a log-normal distribution with the metaparameter θ as follows:

$$\begin{array}{l} \sigma_{k}^{2}\sim \text{LogN}\left(-0.7/\theta ,\;0.7\theta \right); \end{array}$$

and ε_*ij, k*_ is the noise, as defined below. The distribution of $\sigma_{k}^{2}$ resulted that the hidden-layer neurons ranged from neurons that responded to specific input to neurons that responded to a wide range of input. This setting is consistent with physiological observations (Bordi and LeDoux, 1992) and underlies generalization in OVaRLAP. We set the metaparameter θ to regulate the amount of generalization. The noise ε_*ij, k*_ is unnecessary in normal cases; however, we introduced it for the purpose of analyzing the effect of abnormal perturbation in OVaRLAP (Figure 1C). When simulating abnormal perturbation, we applied noise ε_*ij, k*_ by using the following formulas:

$$\begin{array}{l} P\left(\varepsilon_{ij,k}=A\right)=\rho \end{array}$$

and

$$\begin{array}{l} P\left(\varepsilon_{ij,k}=0\right)=1-\rho . \end{array}$$

*A* denotes the noise strength. Apparently, no noise exists if *A* or ρ is zero. This noise was initially introduced but not changed through the learning process. In our simulation experiment, we applied different realizations of noise ε_*ij, k*_ under specific values of *A* and ρ, and examined the collective behaviors of the learning.

The two pre-output neurons then received signals from the hidden-layer neurons. For *m* = 1, 2, the activities of the pre-output neurons *d*_*m*_(*x, y, t*) are given by

$$\begin{array}{l} d_{m}\left(x,y,t\right)=\underset{k}{\sum }w_{m,k}\left(t\right)h_{k}\left(x,y,t\right), \end{array}$$

in which *w*_*m, k*_(*t*) is the weight of the connection from hidden-layer neuron *k* to the pre-output neuron *m* for *m* = 1, 2 and *k* = 1, …, 900. In this instance, *d*_1_(*x, y, t*) and *d*_2_(*x, y, t*) represent the positive and negative values, respectively. The final output of the network *v*(*x, y, t*) integrated these values, as follows:

$$\begin{array}{l} v\left(x,y,t\right)=d_{1}\left(x,y,t\right)-d_{2}\left(x,y,t\right). \end{array}$$

During the interaction between the agent and the environment, the connection weights *w*_*m, k*_(*t*) were updated, depending on the prediction error, δ. To calculate δ, we used an action-value function *Q*(*s, a, t*) for state *s* and action *a* at time *t* (Sutton and Barto, 2018). We defined *Q*(*s, a, t*) by using the output of the network, as follows:

$$\begin{array}{l} Q\left(s,a,t\right)=v\left(x^{\prime },y^{\prime },t\right), \end{array}$$

in which *s* = (*x, y*), *a* = (Δ*x*, Δ*y*), *x*′ = *x* + Δ*x*, and *y*′ = *y* + Δ*y*. In our navigation tasks, (Δ*x*, Δ*y*) = (1, 0), (−1, 0), (0, −1), or (0, 1), if the action was effective. The prediction error δ was represented by

$$\begin{array}{l} \delta \left(t\right)=r\left(s,a\right)+\gamma Q\left(s^{\prime },a^{\prime },t\right)-Q\left(s,a,t\right), \end{array}$$

in which *r*(*s, a*) is the actual reward, *s*′ denotes the state at time *t* + 1, *a*′ denotes the action at time *t* + 1, and γ is the discount factor. We used temporal difference (TD) learning for the action-value function (i.e., state–action–reward–state–action [SARSA]) (Rummery and Niranjan, 1994) because the agent needed to remain at the same square after an ineffective action (i.e., hitting a wall). If we used classical TD learning for the state-value function, such ineffective actions would have disturbed the value learning. The connection weight *w*_1, *k*_(*t*) for *k* = 1, …, 900 was updated only when δ(*t*) was positive, and *w*_2, *k*_(*t*) for *k* = 1, …, 900 was updated only when δ(*t*) was negative. In the actual implementation, the updating rules were as follows:

$$\begin{array}{l} w_{1,k}\left(t+1\right)=\left\{ \begin{array}{c} w_{1,k}\left(t\right)+\frac{\alpha_{1}\delta \left(t\right)h_{k}\left(x^{\prime },y^{\prime },t\right)d_{1}\left(x^{\prime },y^{\prime },t\right)}{N_{1}\left(x^{\prime },y^{\prime },t\right)},\text{if }\delta \left(t\right)>0\\ \;w_{1,k}\left(t\right),\text{ otherwise}, \end{array}\right. \end{array}$$

and

$$\begin{array}{l} w_{2,k}\left(t+1\right)=\left\{ \begin{array}{c} w_{2,k}\left(t\right)+\frac{\alpha_{2}\left[-\delta \left(t\right)\right]h_{k}\left(x^{\prime },y^{\prime },t\right)d_{2}\left(x^{\prime },y^{\prime },t\right)}{N_{2}\left(x^{\prime },y^{\prime },t\right)},\text{if }\delta \left(t\right)<0\\ \;w_{2,k}\left(t\right),\text{ otherwise}, \end{array}\right. \end{array}$$

in which α_1_ and α_2_ are the learning rates and *N*_1_(*x, y, t*) and *N*_2_(*x, y, t*) are the normalization terms, which are represented as

$$\begin{array}{l} N_{m}\left(x,y,t\right)=d_{m}\left(x,y,t\right)\underset{k}{\sum }{h\left(x,y,t\right)}^{2} \end{array}$$

for *m* = 1, 2. These normalization terms are introduced to make sure that.

$$\begin{array}{l} Q\left(s,a,t+1\right)=\left\{ \begin{array}{c} Q\left(s,a,t\right)+\alpha_{1}\delta \left(t\right),\text{if }\delta \left(t\right)>0\\ Q\left(s,a,t\right)+\alpha_{2}\delta \left(t\right),\text{otherwise}. \end{array}\right. \end{array}$$

### Algorithms for Comparison

We also implemented two representative algorithms to compare with the OVaRLAP model. One algorithm was simple TD learning (Sutton and Barto, 2018) (Figure 1A, right top); in our particular case, the algorithm was SARSA. In our implementation, a value function of states, *v*_*s*_(*x, y, t*), was represented as a look-up table and updated as

$$\begin{array}{l} v_{s}\left(x,y,t+1\right)=v_{s}\left(x,y,t\right)+\alpha_{s}\delta_{s}\left(t\right) \end{array}$$

in which α_*s*_ is the learning rate and δ_*s*_ is the prediction error, called the “TD error.” This error is given by

$$\begin{array}{l} \delta_{s}\left(t\right)=r\left(s,a\right)+\gamma_{s}Q_{s}\left(s^{\prime },a^{\prime },t\right)-Q_{s}\left(s,a,t\right) \end{array}$$

in which γ_*s*_ is the discount factor. The action-value function, *Q*_*s*_(*s, a, t*), is given by

$$\begin{array}{l} Q_{s}\left(s,a,t\right)=v_{s}\left(x^{\prime },y^{\prime },t\right). \end{array}$$

The other algorithm is the MaxPain algorithm (Elfwing and Seymour, 2017) (Figure 1A, right bottom). This method is characterized by its distinct systems for the learning values for reward and pain (or punishment). The policy is dependent on the linear combination of the two value functions. In our implementation, we slightly modified the originally proposed MaxPain algorithm to make it comparable with the other methods, while maintaining the essential idea of MaxPain.

First, we used state-value functions of states *v*_*r*_(*x, y, t*) and *v*_*p*_(*x, y, t*), and their linear combination *v*_*L*_(*x, y, t*), to define the action-value functions $Q_{r}\left(s^{\prime },a^{\prime },t\right)$, $Q_{p}\left(s^{\prime },a^{\prime },t\right)$, and $Q_{L}\left(s^{\prime },a^{\prime },t\right)$, as follows:

$$\begin{array}{l} Q_{r}\left(s,a,t\right)=v_{r}\left(x^{\prime },y^{\prime },t\right), \end{array}$$

$$\begin{array}{l} Q_{p}\left(s,a,t\right)=v_{p}\left(x^{\prime },y^{\prime },t\right), \end{array}$$

and

$$\begin{array}{l} Q_{L}\left(s,a,t\right)=v_{L}\left(x^{\prime },y^{\prime },t\right). \end{array}$$

Second, we set the linear combination *v*_*L*_(*x, y, t*) without normalization, as follows:

$$\begin{array}{l} v_{L}\left(x,y,t\right)=v_{r}\left(x,y,t\right)-v_{p}\left(x,y,t\right). \end{array}$$

The couple of state-value functions were implemented as look-up tables and updated as

$$\begin{array}{l} v_{r}\left(x,y,t+1\right)=v_{r}\left(x,y,t\right)+\alpha_{r}\delta_{r}\left(t\right) \end{array}$$

and

$$\begin{array}{l} v_{p}\left(x,y,t+1\right)=v_{p}\left(x,y,t\right)+\alpha_{p}\delta_{p}\left(t\right) \end{array}$$

in which α_*r*_ and α_*p*_ are the learning rates for reward and pain, respectively, and δ_*r*_ and δ_*p*_ are the prediction errors for reward and pain, respectively. The prediction errors were calculated, depending on whether the reward observation was positive or negative, as follows:

$$\begin{array}{l} \delta_{r}\left(t\right)=\varphi \left(r\left(s,a\right)\right)+\gamma_{r}Q_{r}\left(s^{\prime },a^{\prime },t\right)-Q_{r}\left(s,a,t\right) \end{array}$$

and

$$\begin{array}{l} \delta_{p}\left(t\right)=\varphi \left(-r\left(s,a\right)\right)+\gamma_{p}Q_{p}\left(s^{\prime },\mathrm{argmin}\left(Q_{L}\left(s^{\prime },a^{\prime },t\right)\right),t\right)\\ -Q_{p}\left(s,a,t\right) \end{array}$$

in which φ(*z*) = max(*z*, 0) and γ_*r*_ and γ_*p*_ are the discount factors. A variant of off-policy Q-learning algorithm was used to calculate the pain prediction error δ_*p*_, which enabled *Q*_*p*_ to learn for maximizing future pain (i.e., for predicting the worst case). These update rules were the same as those in the original study.

### Action Selection

We used the softmax behavioral policy consistently for the three methods. It depends on the value function ṽ(*x, y, t*), i.e., *v*(*x, y, t*) for OVaRLAP, *v*_*s*_(*x, y, t*) for the simple TD learning, and *v*_*L*_(*x, y, t*) for MaxPain. The probability that the agent selects an action *a* at position (*x, y*) at time *t* is given by

$$\begin{array}{l} \pi \left(a|x,y,t\right)=\frac{\exp\left(\tilde{v}\left(x^{\prime },y^{\prime },t\right)/\tau \right)}{\sum_{c}\exp\left(\tilde{v}\left(x_{c},y_{c},t\right)/\tau \right)} \end{array}$$

in which *x*′ and *y*′ denote the new state after selecting action *a*, *x*_*c*_, and *y*_*c*_ denote the new state after selecting one of the possible actions, and τ is the temperature that controls the trade-off between exploration and exploitation. In our implementation, we used the common τ = 0.5, for the three learning methods.

### Painful Grid-World Navigation Task

The purpose of this task was to navigate from the starting position to either of the two goals, while avoiding hitting the wall. Possible actions at each time step were moving one step north, south, east, and west. For example, if the agent moved one step north, (Δ*x*, Δ*y*) = (0, 1). Two goals exist with reward of 1 or 2. If the agent hits a wall, then it received a negative reward of −1 and remained at the same position. This was an exceptional case. In other cases, the agent could by necessity move to the next square. An episode began when the agent started from the starting position and ended when the agent reached either of the goals. The agent repeated such learning episodes.

A single run consisted of 500 learning episodes, after initializing the value function so that the value for each state was zero. We ran 50 separate runs each for OVaRLAP, simple TD learning, and MaxPain with a single grid-world configuration. We conducted five simulation experiments for each of which we used a grid-world with a consistent character but different configuration. The structure of each grid-world is shown in Figure 1B and in Supplementary Figure S1. We tested OVaRLAP with various amount of generalization by setting θ as θ ∈ {0.44, 0.66, 1.0, 1.5, 2.2}. The value of θ was fixed in each simulation experiment. We set *A* = ρ = 0, that is, no noise in the fixed connections in OVaRLAP. We set the other metaparameters for each algorithm as follows: α_1_ = α_2_ = 0.1, and γ = 0.95 for OVaRLAP; α_*s*_ = 0.1 and γ_*s*_ = 0.95 for simple TD learning; and α_*r*_ = α_*p*_ = 0.1, γ_*r*_ = 0.95, and γ_*p*_ = 0.5 for MaxPain. OVaRLAP and MaxPain are extended algorithms from simple TD; therefore, they used common metaparameters with simple TD, when they could share them.

The five configurations in Supplementary Figure S1 were generated, based on the following rules, while avoiding symmetric or similar structures. Each configuration had a wide passage from which a narrow passage branched off. These passages had fixed widths and lengths. The starting position and the goal with a reward of one were at either end of a wide passage. A goal with reward of two was at the end of a narrow passage. The legend for Supplementary Figure S1 provides further details of the rules.

### Grid-World Navigation Task for Disturbed OVaRLAP

The purpose of this task was to navigate from the starting position to either of four goals. The structure of the grid-world is shown in Figure 1D. Possible actions at each time step were moving one step north, south, east, and west. Multiple goals existed, each of which gave a reward of one. Our interest in this experiment was not in pain aversion; therefore, we did not apply a negative reward to the actions that resulted in hitting a wall (i.e., no pain). The agent simply remained at the same position after hitting a wall. Even in this no pain setting, the agents after reinforcement learning likely avoid wall hits, due to the discount factor in the value functions. An episode began when the agent moved from the starting position and ended when the agent reached one of the four goals. A single run consisted of 40,000 time steps in total (i.e., 378.8 learning episodes on average) after the value function initialization at the onset of the first learning episode. For each metaparameter setting described below, we performed 50 separate runs for OVaRLAP. We considered the following cases: normal or no update for a negative TD error (i.e., “intact” or “impaired”) and with or without noise in the fixed connections (i.e., “noised” or “unnoised”). There were 25 different settings for the noised case, consisting of pairs of noise strength *A* and noise fraction ρ, taken from *A* ∈ {0.25, 0.5, 1, 2, 4} and ρ ∈ {0.00125, 0.0025, 0.005, 0.01, 0.02}, respectively. Although the values of *A* and ρ were fixed within each simulation setting, the noise, ε_*ij, k*_, was generated independently for each of the 50 runs and was fixed within each run. We set the other metaparameters, as follows: θ = 1; *A* = ρ = 0 for the unnoised case; α_1_ = 0.1; α_2_ = 0.1 for the intact setting (which was the same as in the previous experiment) and α_2_ = 0 for the impaired setting; γ = 0.8; and τ = 0.5.

## Results

### The Behavior of OVaRLAP

**Figure 2:**
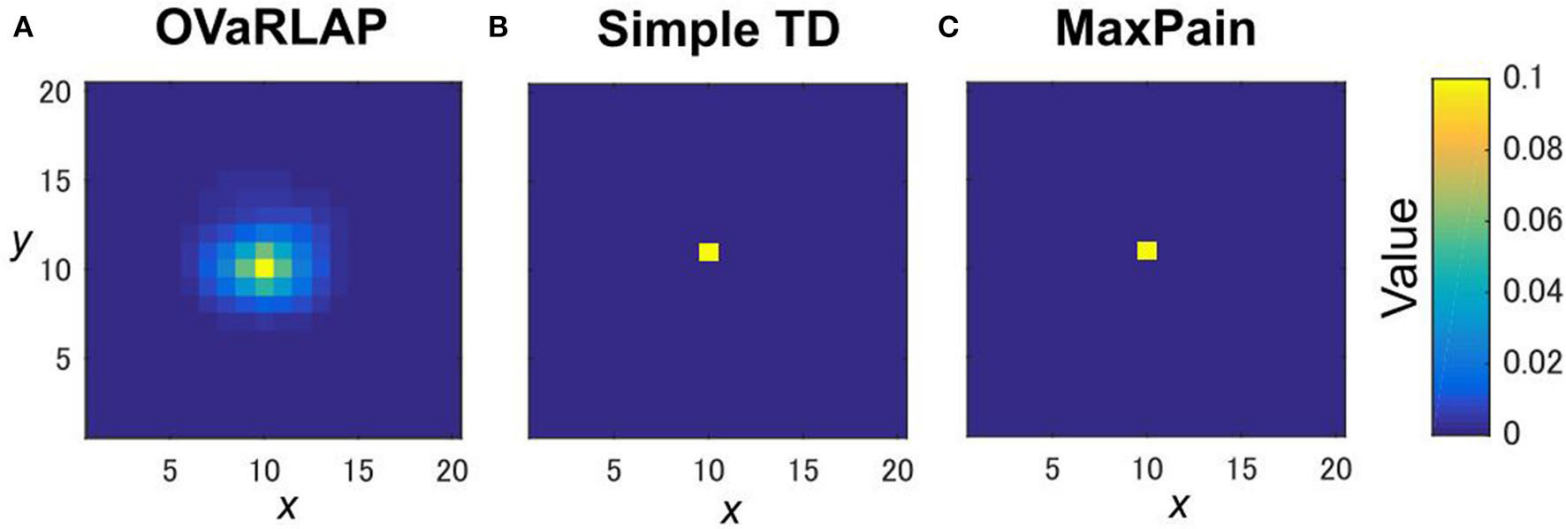
State values after a single update. The value function is updated after receiving a positive reward of one once with OVaRLAP **(A)**, simple TD learning **(B)**, and MaxPain **(C)** at the center of a two-dimensional grid-world in Figure 1B. For OVaRLAP, the metaparameter θ is set as θ = 1 to introduce a moderate amount of generalization.

We evaluated OVaRLAP in a spatial navigation task in painful grid-worlds by comparing its performance with those of simple TD learning and MaxPain (Elfwing and Seymour, 2017). Figure 1A shows a schematic representation of these algorithms. OVaRLAP achieves stimulus generalization based on the similarity of states (Figure 2A) because it utilizes a fixed neural network (gray arrows in Figure 1A, left) for the preprocessing of the value learning network (blue and red arrows in Figure 1A, left). By contrast, the other two algorithms, which have look-up tables, show no stimulus generalization (Figures 2B,C). The task in this study was to seek rewarded goals while avoiding painful wall hits in which the agent had to manage the trade-off between obtaining rewards and avoiding pain (Figure 1B). We used five different grid-worlds, each of which had a safe but lowly rewarded goal and a risky but highly rewarded goal (Supplementary Figure S1).

**Figure 3:**
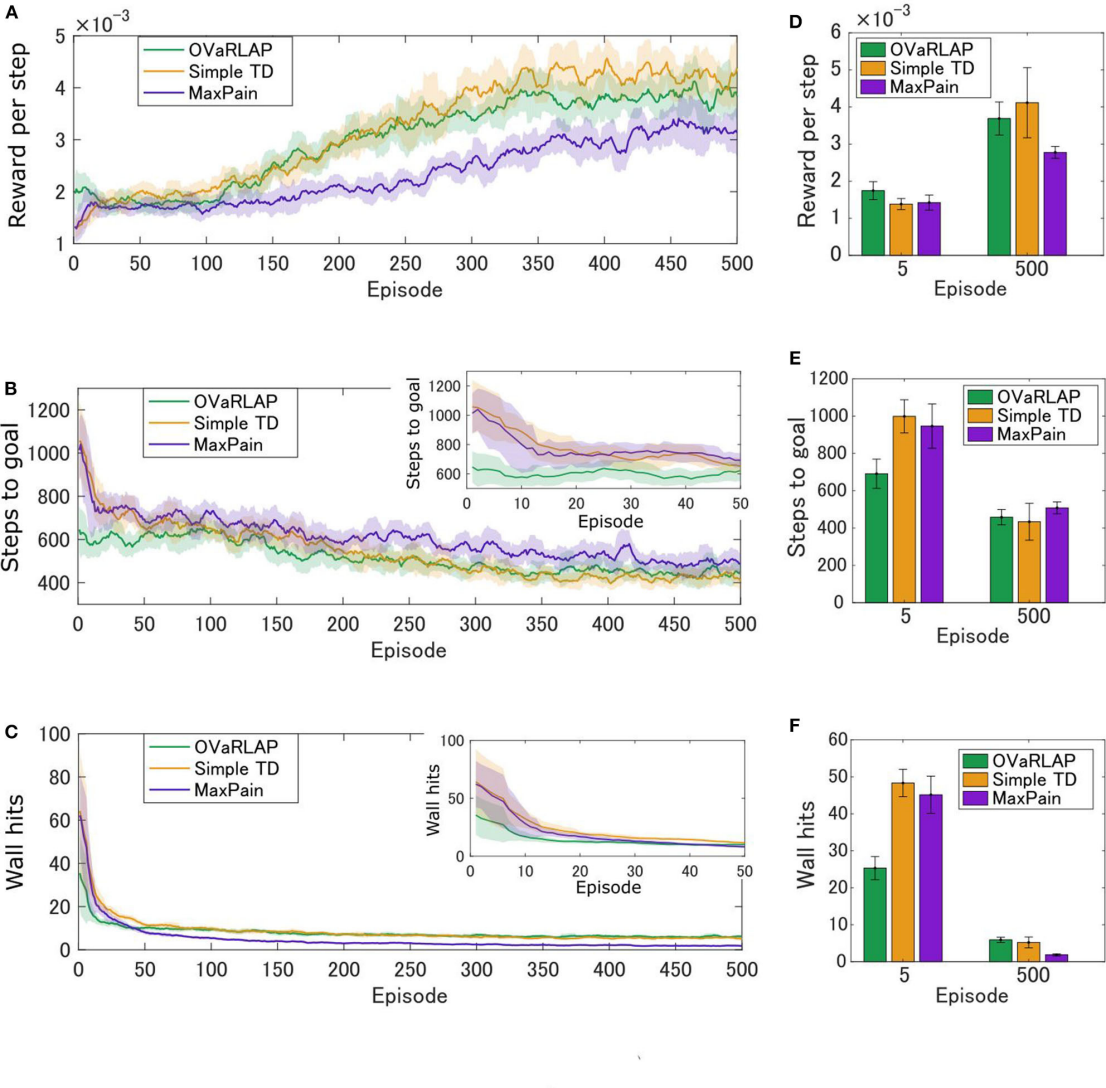
Average performance of OVaRLAP, the simple TD learning, and MaxPain in the painful grid-world navigation task. **(A)** The average reward per step, **(B)** the average number of steps to either of the two goals, and **(C)** the average number of wall hits, based on the three types of agents after each number of learning episodes on the horizontal axis. The grid world shown in Figure 1B was used. The number of steps to either of the two goals and the number of wall hits for the first 50 learning episodes are magnified in the top right insets of **(B,C)**, respectively. Each average (on the vertical axis) was obtained over 50 separate runs and then smoothed using a moving average over the preceding 11 episodes (on the horizontal axis). The thick lines represent the moving averages and the shadow areas indicate the moving standard deviations. **(D–F)** The average performance over five simulation experiments, each of which used a grid-world with different configurations. The grid world shown in Figure 1B and its variants were used (Supplementary Figure S1 presents the details of the variants). **(D)** The average reward per step, **(E)** the average number of steps to either of the two goals, and **(F)** the average number of wall hits, based on the three types of agents after five learning episodes and 500 learning episodes, are shown. Each bar indicates the average over five grid-world configurations, after taking the average over 50 separate runs with each grid-world configuration. Each error bar represents the standard deviation over the five configurations. For OVaRLAP, the metaparameter θ was set as θ = 1 to induce a moderate amount of generalization.

**Figure 4:**
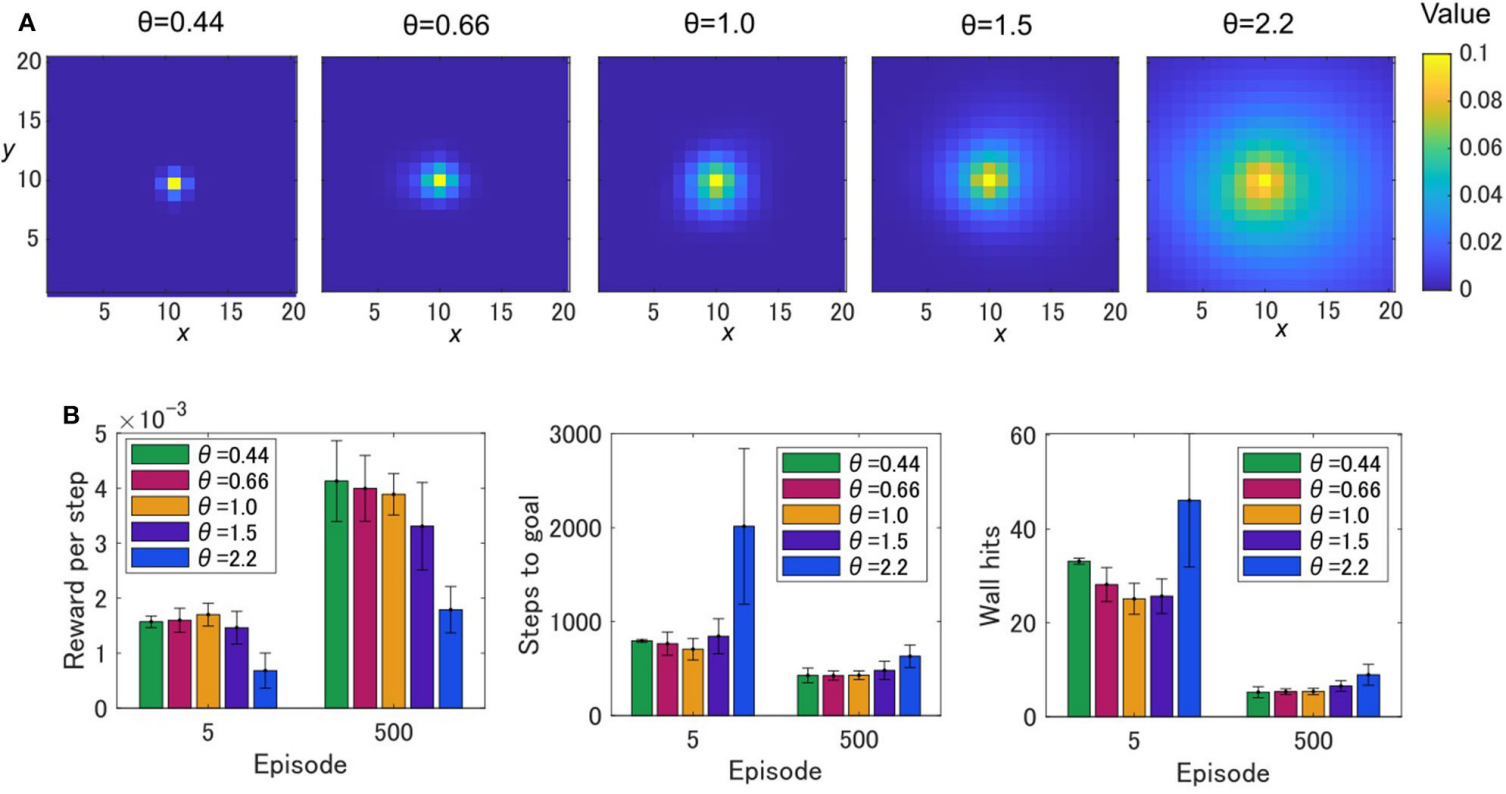
The painful grid-world navigation task for OVaRLAP with various amount of generalization. **(A)** State values after a single update for OVaRLAP with a variety of metaparameter settings, θ ∈ {0.44, 0.66, 1.0, 1.5, 2.2}, which control the level of generalization. The value function is updated after receiving a positive reward of one once at the center of a two-dimensional grid-world in Figure 1B. **(B)** The average performance over five simulation experiments, each of which used a grid-world with a different configuration than the others. The grid-world shown in Figure 1B and its variants were used (Supplementary Figure S1 presents the details of the variants). The average reward per step (left), the average number of steps to either of the two goals (middle), and the average number of wall hits (right), over five OVaRLAP agents with different metaparameter settings, θ ∈ {0.44, 0.66, 1.0, 1.5, 2.2}, after five learning episodes and 500 learning episodes, are shown. Each bar indicates the average over five grid-world configurations, after taking the average over 50 separate runs with each grid-world configuration. Each error bar represents the standard deviation over the five configurations.

Figures 3A–C show the average learning curves at each episode over 50 independent runs for the grid-world shown in Figure 1B. The reward per step of the OVaRLAP agent was higher than that of the MaxPain agent, but it did not exceed that of the simple TD learning agent (Figure 3A). Compared to the other agents, the OVaRLAP agent reached each of the goals with a smaller number of steps in the early learning phase (Figure 3B). After 100 learning episodes, all three agents showed a similar number of steps to reach either of the goals. With regard to pain aversion, the OVaRLAP agent also showed quick learning. The OVaRLAP agent exhibited fewer wall hits in the early learning phase than the other agents did. However, after 50 learning episodes, the number of wall hits of the OVaRLAP agent was comparable to that of the simple TD learning agent (Figure 3C). By contrast, the MaxPain agent first hit the walls as many times as the simple TD learning agent, but the number of wall hits then quickly decreased. The OVaRLAP agent showed its characteristic performance in all of the five simulation experiments, each of which had a different grid-world with a similar spatial structure (Figures 3D–F). The relative performance of the three agents was consistent when the starting position was randomized (Supplementary Figure S2). We also tested the OVaRLAP agents with various levels of generalization (Figure 4A). The results showed that an increase in generalization led to better performance unless the generalization became too large, whereas too large generalization deteriorated the performance (Figure 4B). These results suggest that the OVaRLAP agent could learn a reward approach and pain aversion in a very efficient manner owing to its proper generalization, whereas it was inferior to the simple TD in long-term reward learning and to MaxPain in long-term pain aversion.

**Figure 5:**
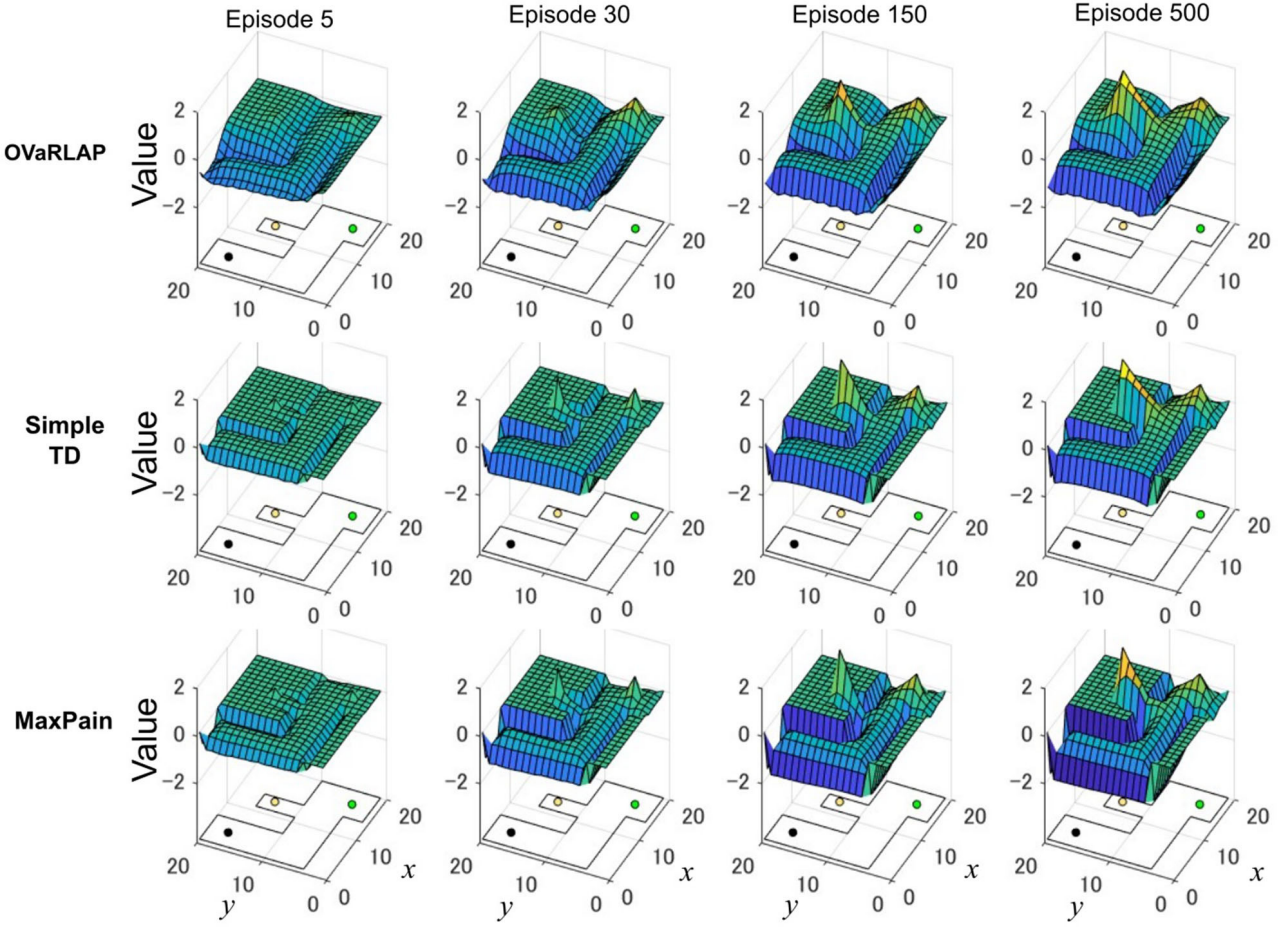
Transition of the value function for each model in the grid-world navigation task. The average state values over 50 separate runs after 5, 30, 150, and 500 learning episodes for OVaRLAP (top row), simple TD learning (middle row), and MaxPain (bottom row), when applied to the painful grid-world navigation task shown in Figure 1B. An outline of the grid-world is shown on the floor of each plot. The black lines indicate walls. The black, green, and yellow circles are the starting position, the low-reward goal, and the high-reward goal, respectively. For OVaRLAP, the metaparameter θ is set as θ = 1 to introduce a moderate level of generalization. For MaxPain, the result of the subtraction of the value function for the pain from the value function for the positive reward (i.e., the main factor for decision-making) is displayed.

Figure 5 shows how the value function developed using the three methods, in which OVaRLAP exhibited unique profiles (Figure 5, top). First, the values of positions close to the walls decreased, as did the values of the positions of the walls. After only five learning episodes, this generalization constructed a safe passage to the low-reward goal while simultaneously causing a dip in the value function as a hazard to approach the risky but high-reward goal. This value hazard completely disappeared after 500 episodes because the reward learning had propagated to the squares along the passage to the high-reward goal. Thus, quick pain learning was essential for aggressive pain aversion and conservative reward-seeking in the early learning stage.

The simple TD learning algorithm did not construct any value hazard to the high-reward goal during its learning process because no special system existed for pain learning (Figure 5, middle). The MaxPain algorithm also produced hazards on the passage to the high-reward goal (Figure 5, bottom). In contrast to OVaRLAP, the hazard progressively increased as the learning proceeded and never flattened owing to its strong pain learning ability. For this reason, the MaxPain algorithm persistently maintained low values on the way to the high-reward goal.

**Figure 6:**
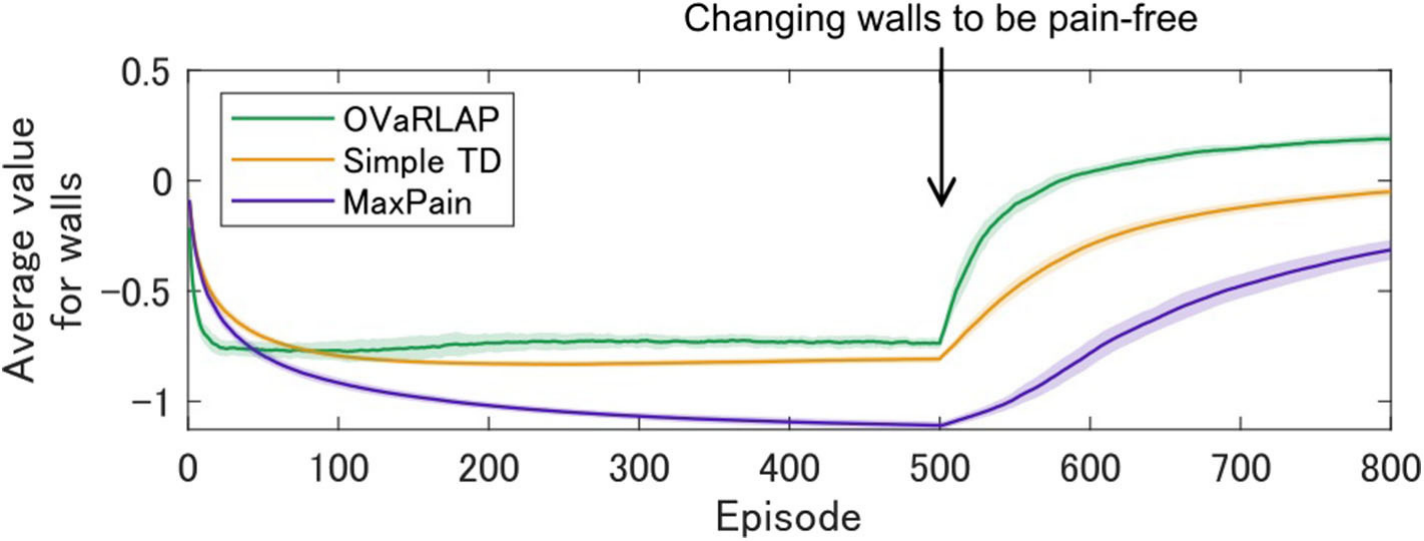
Relearning values of walls after environmental changes. OVaRLAP, the simple TD learning, and MaxPain were applied to a grid-world navigation task in which the agent was trained with painful walls until the 500th episode and the environment was changed at the 500th episode to have pain-free walls (other than walls, everything was the same before and after the change). The grid-world shown in Figure 1B was used. The state-value averaged over all wall positions is plotted for each of the three agents, on the horizontal axis indicating the number of learning episodes. Each average (on the vertical axis) is obtained over 50 separate runs and the shadow areas indicate the standard deviation. For OVaRLAP, the metaparameter θ is set as θ = 1 to introduce a moderate level of generalization.

We further evaluated how the model agent navigates if the environment is changed to have pain-free walls after the agent has been trained with painful walls (other than walls, everything is the same before and after the change). Interestingly, the OVaRLAP agent quickly unlearnt the past painful stimuli, compared to the other two models (Figure 6). This result indicates that generalization worked not only in the early phase of learning but also contributed to relearning timings induced by environmental changes.

### The Effect of a Disturbed OVaRLAP

**Figure 7:**
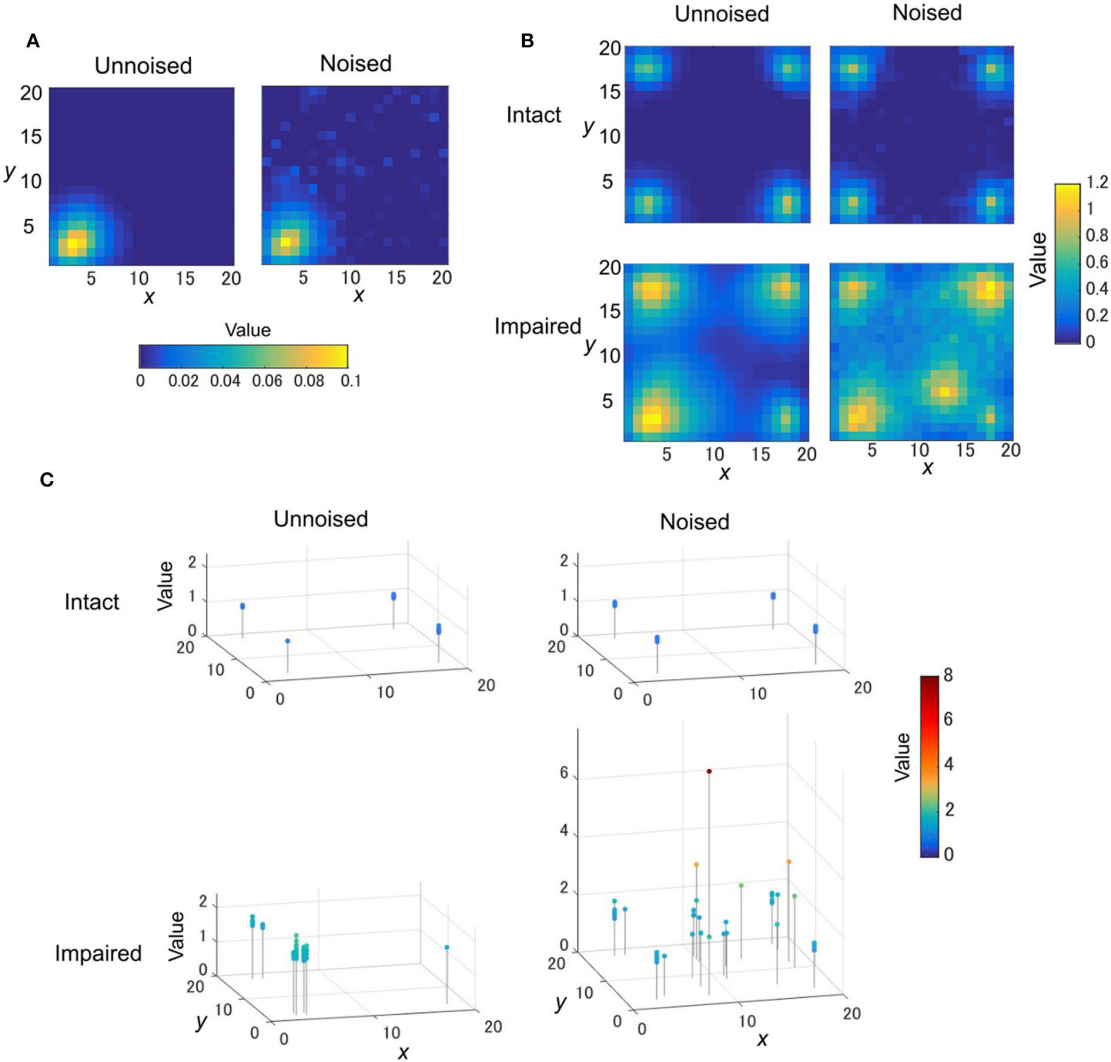
The TD learning task for disturbed OVaRLAP. **(A)** The value function is updated after reaching the left-bottom goal of the grid-world shown in Figure 1D once with OVaRLAP without noise (left) and with noise (right). **(B)** The value function after 120 learning episodes in a single run for OVaRLAP with intact and impaired updates (top and bottom rows, respectively) for the negative TD error with and without noise (right and left columns, respectively) in the fixed connections. **(C)** The maximum value and its position in the value function after 40,000 time steps in total (378.8 learning episodes on average) in each run. Each panel shows the maximum values in 50 separate runs for OVaRLAP under the following settings: intact or impaired updates (top or bottom row, respectively) for the negative TD error, and without noise or with noise (left or right column, respectively) in the fixed connections. In the noisy case, the noise was independently introduced to the initialization of the fixed connections for each run.

We next examined how the learning behaviors of OVaRLAP were disturbed by disabling weight updating by negative TD errors and introducing noise to the fixed network (Figure 1C). The generalization after obtaining a reward became a little noisy because of the noise introduction (Figure 7A). We applied this disturbed OVaRLAP to a grid-world navigation task without pain (Figure 1D). We tested each of the following two-by-two settings: normal or no update for the negative TD error (i.e., “intact” or “impaired”), and with or without noise in the fixed network (i.e., “noised” or “unnoised”).

The impaired noised agent acquired an aberrant value function: it increased the value for a specific position distant from the goals, as if the position would give a positive reward (Figure 7B, bottom right). The value function of the intact noised agent was slightly noisy (Figure 7B, top right), but nearly the same as that of the intact unnoised agent (Figure 7B, top left). The impaired unnoised agent made the values around the goals higher than those of the intact agents, but the values for positions distant from the goals remained low (Figure 7B, bottom left).

We ran 50 runs for each setting to confirm reproducibility. For the noised agent, we applied a different pattern of noise in each run. Figure 7C presents the maximum value and its position in the value function after 40,000 steps in each run. The intact agents always established the maximum values at the positions of goals and maintained them consistent to the actual amount of the rewards the agent obtained (Figure 7C, top row). The maximum values of the impaired unnoised agent were higher than the actual amount of the rewards (Figure 7C, bottom left). Their positions were not necessarily the same as the actual goals, but they were, at most, two steps away from the goals. In contrast to these agents, the impaired noised agent produced maximum values that were quite apart from the actual goals (Figure 7C, bottom right). These values were higher than the actual amount of rewards and were sometimes exceedingly high.

For the noised case, we tested different noise settings by varying the noise strength and the noise fraction (Supplementary Figure S3), and for each we performed 50 runs. Supplementary Table S1 provides a summary of the results and shows that the various levels of noise induced aberrant valuation if the negative value neuron was impaired. It also shows that sole large noise did not lead to aberrant valuation as long as the negative value learning was intact.

## Discussion

### The Effect of Generalization and Discrimination on Behaviors

In this study, we proposed a novel reinforcement learning model named OVaRLAP to analyze how the computational characteristics in the striatal dopamine system affect behaviors. The OVaRLAP model reproduced stimulus generalization for both reward learning and punishment learning. Discrimination followed generalization to shape the value function, that is, inhibiting excessive expectation of reward or punishment caused by generalization. It was realized by the activity of one pre-output neuron that offset the activity of the other pre-output neuron. In the navigation task in painful grid-worlds (Figures 3–5), punishment learning due to painful wall hits was first generalized and subsequent unexpected safeness caused discrimination. By contrast, in the case of painless grid-world (Figure 7), generalization for reward learning was followed by discrimination due to the unexpected unrewarded outcome.

The OVaRLAP model enabled safe and efficient exploration in the painful grid-world navigation task. The transition of the value function for the OVaRLAP agent shows how stimulus generalization and discrimination contribute to managing the trade-off between reward-seeking and pain aversion (Figure 5, top).

First, punishment learning due to hitting walls generalized quickly, as shown in the value function after five episodes. It led to a preference for the center of the passage (i.e., pain aversion). Indeed, the OVaRLAP agent first showed fewer wall hits than the simple TD learning and MaxPain agents did (Figures 3C,F). This contributed to reducing the number of steps to reach either of the goals in the early learning phase (Figures 3B,E). Thereafter, The OVaRLAP agent increased the values of positions close to the walls. This transition indicated that the agent discriminated between the safe and painful positions. This discrimination increased the tendency to reach the high-reward goal. In short, stimulus generalization of punishment learning induced pain aversion, followed by discrimination for switching to reward-seeking. The simple TD learning agent did not show pain aversion (Figure 5, middle). Its value function was optimized to maximize future reward, which is consistent with its high reward per step (Figures 3A,D). The MaxPain agent showed strong pain aversion based on its separate value learning system to expect future pain (Figure 5, bottom row). It achieved very few wall hits (Figures 3B,E) while maintaining pain aversion and low values on the way to the high-reward goal, and this corresponded to a lower reward per step compared to the other agents (Figures 3A,D).

Stimulus generalization and discrimination were effective in safe and efficient exploration; however, dysfunction in the system may induce aberrant learning behaviors. First, too large of a generalization deteriorated the performance of the OVaRLAP agent (Figure 4), which is consistent with the previous reports that generalization to an abnormal extent is associated with psychiatric disorders (Dunsmoor and Paz, 2015; Asok et al., 2018). Second, the impaired update for the negative TD error combined with the noise in the fixed connections induced aberrant valuation in OVaRLAP (Figure 7, Supplementary Table S1). The value increased by noisy stimulus generalization was not reshaped by discrimination because of the impairment of the punishment learning. A single update of the value was only slightly affected by the noisy stimulus generalization (Figure 7A); however, as the number of arrivals to the actual goals increased, such aberrant updates of the value function would have allotted abnormally high values to some positions that were actually of no reward (Figures 7B–C).

### Computation Underlying Generalization

The structure of OVaRLAP provides insight into the neurobiological basis of stimulus generalization and discrimination. The two pre-output neurons separately responded to positive and negative prediction errors to update the connections between the hidden-layer neurons and the corresponding pre-output neuron. This hybrid learning system exhibits good correspondence to the striatal dopamine system in which D1- and D2-SPNs differentially respond to dopamine so that the connections between the cortex and the striatal SPNs are differently modulated (Reynolds et al., 2001; Hikida et al., 2013; Yagishita et al., 2014). Transforming the input into the hidden-layer activity approximates the process by which an external stimulus and the internal state are encoded into neural activity patterns of the cortex. This process is assumed to be rather independent from dopamine-dependent plasticity. However, this process should not be based simply on random connections because the similarity of the inputs has to be maintained in the encoding process to achieve stimulus generalization. Instead, encoding could be learned in a different manner from the reward-related learning. Therefore, the fixed connections between the input and hidden layers in OVaRLAP can be the result of learning for such encoding process. How the brain learns the encoding process is a topic for future research, but some implications are derived from computational models of the primary visual cortex and the primary auditory cortex that adopted unsupervised learning (Hyvärinen and Hoyer, 2001; Terashima and Okada, 2012).

The encoding process may also depend on additional functions other than learning. It has been reported that the prefrontal dopamine system could be involved in processing incoming sensory signals such as working memory and attention (Ott and Nieder, 2019). Although we have not modeled the prefrontal dopamine system in the current OVaRLAP model, one possible implementation would allow prefrontal dopamine to modify the information transmission from the input layer to the hidden layer and thereby alter generalization.

Compared to ordinary reinforcement learning algorithms, the uniqueness of the OVaRLAP model is that it is characterized by fast generalization and separation of positive and negative value learning. Its advantages in safe and efficient exploration primarily appeared due to fast generalization, whereas its potential defect in causing maladaptive behaviors was attributed to fast generalization and also separation of positive and negative value learning. Mikhael and Bogacz previously demonstrated the advantage of separation of positive and negative values (Mikhael and Bogacz, 2016). Their model coded reward uncertainty into the sum of synaptic weights of D1- and D2-SPNs. In addition, their model could adjust for the tendency to choose or avoid risky options by changing the weight of the positive and negative values, which is assumed to reflect tonic dopamine levels. This model is consistent with risk-taking behaviors observed in patients of Huntington's disease, in which striatal neurons are closely involved (Kalkhoven et al., 2014). To further discuss advantages of separation of positive and negative values is beyond the scope of our current study, although the OVaRLAP model may also exhibit such advantages. The OVaRLAP model can encode reward uncertainty in distinct connections updated by positive and negative TD errors (red and blue arrows, respectively, in Figure 1A, left), and can adjust a risk-taking tendency by changing the connection weights between the pre-output neurons to the output neuron, which were set as constants for simplicity in the current study.

### Hypotheses on Psychiatric Disorders

The potential defect of OVaRLAP that causes maladaptive behaviors could provide a hypothesis on psychiatric disorders. Based on the correspondence between OVaRLAP and the brain, our results with the disturbed OVaRLAP showed the possibility that the noisy encoding process in the cortex and the impairment of dopamine-dependent plasticity induce abnormal stimulus generalization and discrimination, which may underlie delusional symptoms. The relationship between cortical disconnectivity and disrupted learning in schizophrenia has been implied in computational studies (Hoffman and Dobscha, 1989; Hoffman and McGlashan, 1997); therefore, alterations in cortical connectivity could be a cause of a noisy encoding process. Histological examinations and diffusion tensor imaging studies of patients with schizophrenia revealed reduced dendritic spine density and disrupted white matter connectivity, respectively (Garey et al., 1998; Ellison-Wright and Bullmore, 2009; van den Heuvel et al., 2016). Reinforcement learning models have been used for attempts to explain positive symptoms, including delusions, in schizophrenia (Deserno et al., 2013; Katahira and Yamashita, 2017; Maia and Frank, 2017). However, these studies do not link abnormal cortical connectivity to positive symptoms. Rather, they attribute positive symptoms to aberrant salience (i.e., a surprise response to non-salient events) (Kapur, 2003). Thus, they focused on abnormalities in the dopamine system, which was also supported by neurobiological evidence (Howes et al., 2012; Daberkow et al., 2013). The OVaRLAP model is a new corticostriatal learning model that relates delusional symptoms to abnormalities in cortical connectivity and the dopamine system.

The relationship between abnormal stimulus generalization and schizophrenia has been investigated in psychological research (Buss and Daniell, 1967; Ralph, 1968; Kahnt and Tobler, 2016) based on the hypothesis that abnormal generalization underlies delusional symptoms in schizophrenia. Some studies imply heightened stimulus generalization in schizophrenia (Ralph, 1968; Kahnt and Tobler, 2016). However, the reported abnormality was not sufficiently remarkable for explaining delusion in a straightforward manner. This could be attributed to the conditioning paradigm used for their behavioral experiments. Patients with schizophrenia have various deficits in cognitive function; therefore, the experimental design needs to be simple to attribute the result to stimulus generalization rather than other factors. Computational models may potentially be used to investigate how a specific deficit in cognitive function leads to psychotic symptoms through complex learning processes. The OVaRLAP model showed that a small abnormality in stimulus generalization in combination with an unbalanced response to positive and negative prediction error may induce aberrant valuation after reward-related learning, including action selection, state transition, and obtaining rewards from multiple sources.

### Conclusions

The OVaRLAP model updates synaptic weights of the last layer in a multilayer neural network, which reflects dopamine-dependent plasticity of corticostriatal synapses (Reynolds et al., 2001). In contrast to ELM (Huang et al., 2006), where fixed connections between the input and hidden layers are set randomly, we set the fixed connections of the OVaRLAP model to maintain the similarity of inputs in the hidden-layer representation. The OVaRLAP model enabled fast generalization of reward and punishment learning. In the painful grid-world navigation tasks, it demonstrated a quick reward approach and efficient pain aversion in the early learning phase and achieved safe and efficient exploration. However, disturbances of the OVaRLAP network that caused noisy generalization and impaired discrimination led to aberrant valuation.

These results suggested the advantage and potential drawback of generalization by the striatal dopamine system with regard to adaptive behaviors. These results are consistent with previous theories in behavioral science (Dunsmoor and Paz, 2015; Kahnt and Tobler, 2016; Asok et al., 2018), in which generalization is considered to be adaptive, whereas abnormal generalization is implicated in maladaptive behaviors. The OVaRLAP model also gives insight into the neurobiological basis of generalization and its dysfunction. The process for encoding external stimuli and internal states into neural activity patterns of the cortex may be learned independently from reward-related learning. Disruption of this encoding process induced by altered cortical connectivity may disturb reward- and punishment-related learning, possibly underlying delusional symptoms of psychiatric disorders.

## Data Availability Statement

The datasets generated for this study are available on request to the corresponding author.

## Author Contributions

YF, SY, HK, and SI conceived the study. YF developed, analyzed, and simulated the models. YF and SI wrote the paper. SY and HK reviewed drafts of the paper. All authors contributed to the article and approved the submitted version.

## Conflict of Interest

The authors declare that the research was conducted in the absence of any commercial or financial relationships that could be construed as a potential conflict of interest.
