# Supplementary material for: Computational Characteristics of the Striatal Dopamine System Described by Reinforcement Learning With Fast Generalization
Source: Front Comput Neurosci. 2020 Jul 22;14:66. doi: 10.3389/fncom.2020.00066 (PMC7388898; doi:10.3389/fncom.2020.00066)
Supplement: Supplementary file 1 [file Data_Sheet_1.PDF]

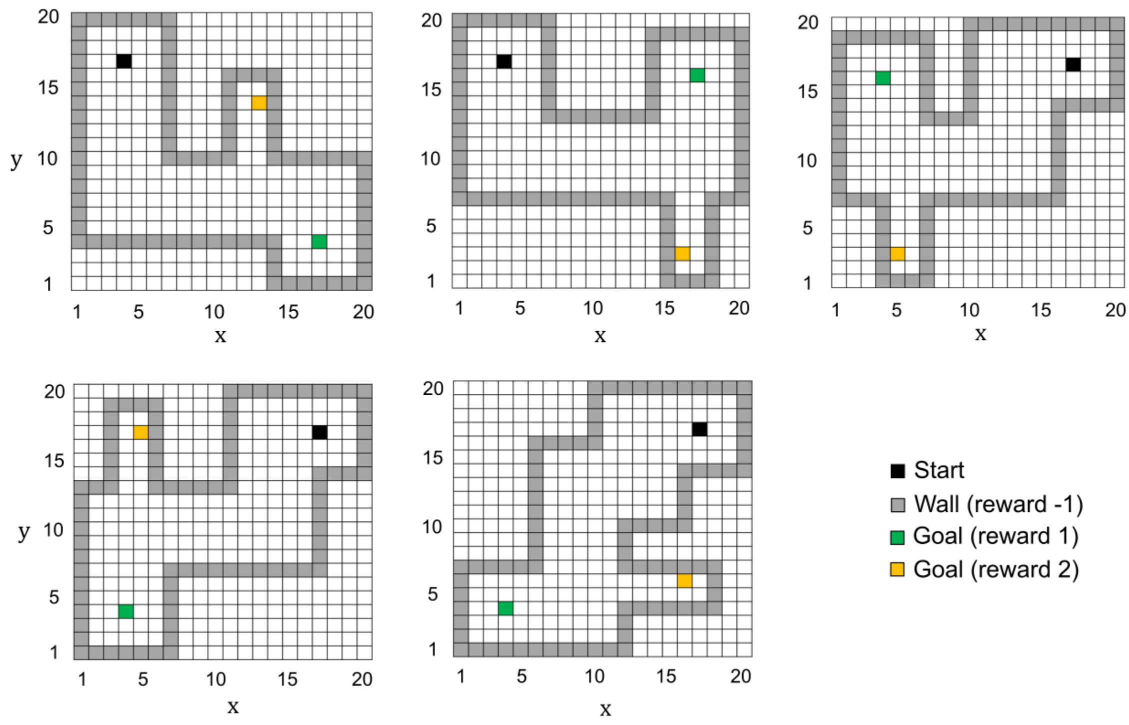

Supplementary Figure S1. The grid-world configurations used in the painful grid-world navigation task. The grid-world shown in Figure 1B is displayed again at the left top. These five grid-world configurations were generated under the following rules, while avoiding symmetrical or similar structures. A wide passage consists of rectangles in which the shorter width/height is 5 (based on the number of grids) and the longer width/height is more than 7; the wide passage does not have a dead end. A narrow passage exists with a width of 2 and a length of 6. The starting position and a goal with a lower reward of 1 are located two grids away from the end of the wide passage and three grids away from side walls (i.e., relatively apart from the walls). The passages have to be surrounded by walls. If walls separate the passages, the distance between these passages has to be more than three grids. A goal with a higher reward of 2 is one grid away from the end of the narrow passage (i.e., relatively close to the walls). The shortest path to either of the goal requires 26 steps if the agent has to move to the center of the wide passage before turning to the narrow passage.

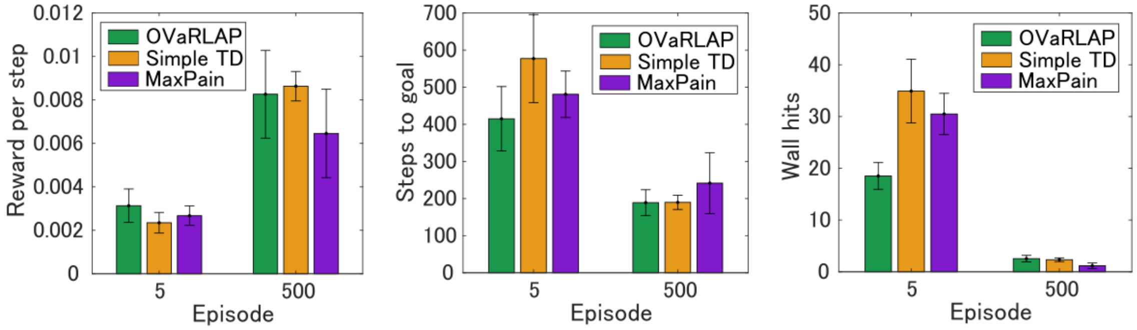

Supplementary Figure S2. Average performance of OVaRLAP, the simple TD learning, and MaxPain in the painful grid-world navigation task with randomized starting positions. The average performance over five simulation experiments, each of which used a grid-world with different configurations. The grid-world shown in Figure 1B and its variants were used (Supplementary Figure S1 presents the details of the variants), but the starting position was randomized for each learning episode. The average reward per step (left), the average number of steps to either of the two goals (middle), and the average number of wall hits (right), after 5 and 500 learning episodes, are shown. Each bar indicates the average over five different grid-world configurations, after taking the average over 50 separate runs for each configuration. Each error bar represents the standard deviation over the five configurations. For OVaRLAP, the metaparameter θ is set as $\theta = 1$ to induce a moderate level of generalization.

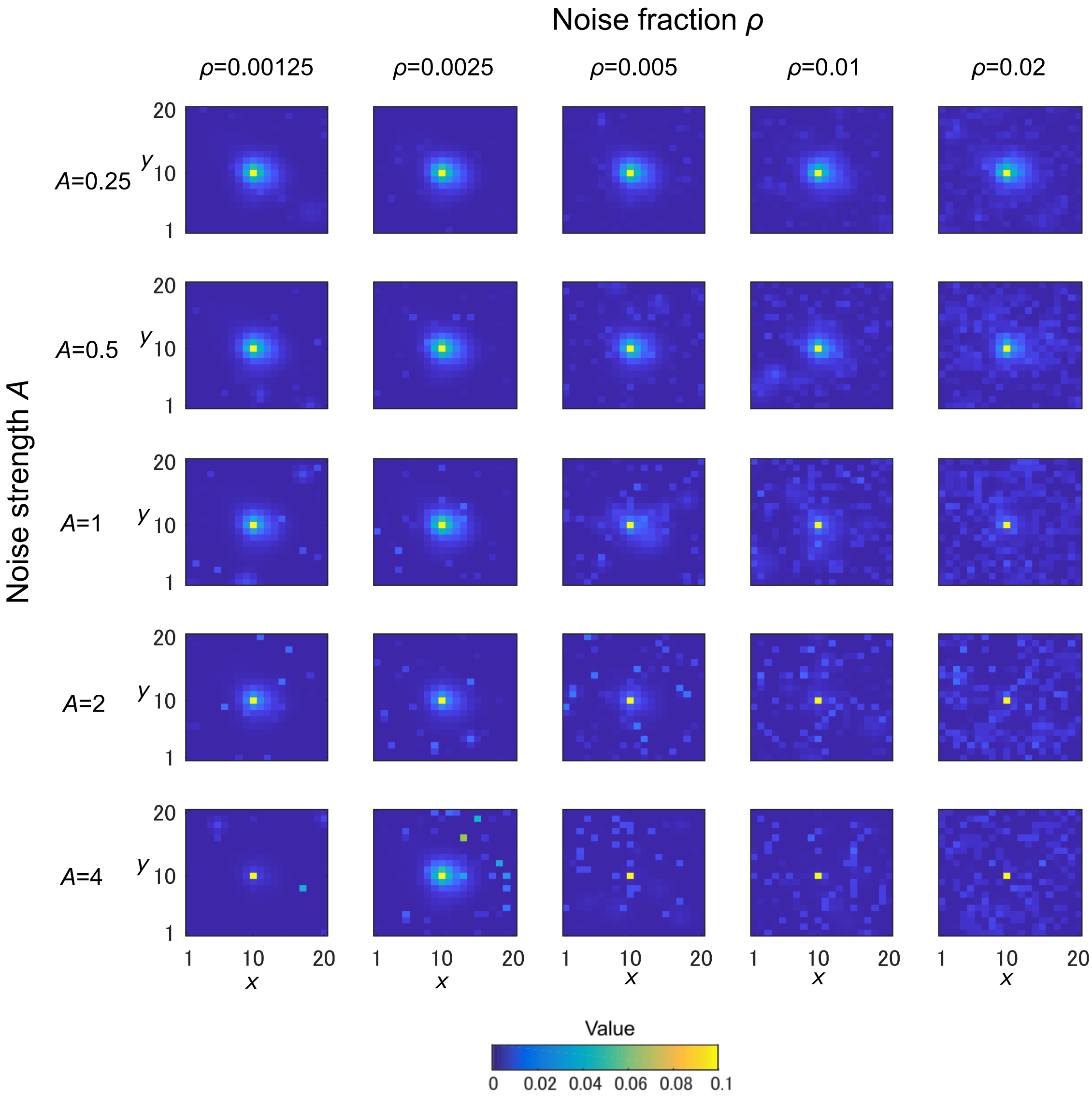

Supplementary Figure S3. State values after a single update for OVaRLAP with various noise settings. State values after a single update for OVaRLAP with various combinations of the metaparameters A and ρ, which denote the noise strength the noise fraction, respectively. These metaparameters were set as $A \in \{0.25, 0.5, 1, 2, 4\}$ and $\rho \in \{0.00125, 0.0025, 0.005, 0.01, 0.02\}$. The value function for each agent was updated after receiving a positive reward of 1 once at the center of a two-dimensional grid-world (shown in Figure 1D).

		Intact			Impaired		
ρ	A	Total runs	Runs causing maximum value at passable grids different from goals	Average of maximum value	Total runs	Runs causing maximum value at passable grids different from goals	Average of maximum value
0.00125	0.25	50	0	1.036	50	24	1.976
	0.5	50	0	1.023	50	29	5.505
	1	50	0	1.029	50	49	24.53
	2	50	0	1.034	50	50	13.23
	4	50	0	1.044	50	49	15.86
0.0025	0.25	50	0	1.031	50	24	1.852
	0.5	50	0	1.027	50	38	5.458
	1	50	0	1.036	50	46	10.57
	2	50	0	1.028	50	49	21.68
	4	50	0	1.026	50	50	15.14
0.005	0.25	50	0	1.033	50	27	2.779
	0.5	50	0	1.027	50	40	4.254
	1	50	0	1.028	50	50	6.351
	2	50	0	1.016	50	50	5.587
	4	50	0	1.005	50	48	8.693
0.01	0.25	50	0	1.021	50	22	4.682
	0.5	50	0	1.027	50	37	3.899
	1	50	0	1.022	50	44	2.668
	2	50	0	1.004	50	48	3.449
	4	50	0	0.9929	50	44	2.923
0.02	0.25	50	0	1.027	50	16	1.781
	0.5	50	0	1.016	50	27	1.909
	1	50	0	1.012	50	35	2.252
	2	50	0	0.9984	50	33	2.338
	4	50	0	0.9893	50	28	2.224

Supplementary Table S1. The TD learning task for disturbed OVaRLAP with various noise settings. When various levels of noise are introduced to the fixed connections, we evaluate the performance of OVaRLAP with intact and impaired updates for the negative TD error. The result of 50 separate runs for each setting of the OVaRLAP agent is shown. The metaparameters A and ρ denote the noise strength and the noise fraction, respectively. The grid-world shown in Figure 1D was used. The noise was independently introduced to the initialization of the fixed connections for each run.
